# Preparation, Characterization, and* In Vivo* Pharmacoscintigraphy Evaluation of an Intestinal Release Delivery System of Prussian Blue for Decorporation of Cesium and Thallium

**DOI:** 10.1155/2017/4875784

**Published:** 2017-11-29

**Authors:** Nidhi Sandal, Gaurav Mittal, Aseem Bhatnagar, Dharam Pal Pathak, Ajay Kumar Singh

**Affiliations:** ^1^Department of Nuclear Medicine, Institute of Nuclear Medicine and Allied Sciences, Defence R&D Organisation, Brig. SK Mazumdar Road, Delhi 110 054, India; ^2^Division of Pharmaceutical Chemistry, DIPSAR, MB Road, Pushp Vihar, New Delhi 110 017, India

## Abstract

**Background:**

Prussian blue (PB, ferric hexacyanoferrate) is approved by US-FDA for internal decorporation of Cesium-137 (^137^Cs) and Thallium-201 (^201^Tl).

**Aim:**

Since PB is a costly drug, pH-dependent oral delivery system of PB was developed using calcium alginate matrix system.

**Methods:**

Alginate (Alg) beads containing PB were optimized by gelation of sodium alginate with calcium ions and effect of varying polymer concentration on encapsulation efficiency and release profile was investigated. Scanning electron microscopy (SEM) was carried out to study surface morphology. Adsorption efficacy of Alg-PB beads for ^201^Tl was evaluated and compared with native PB.* In vivo* pH-dependent release of the formulation was studied in humans using gamma scintigraphy.

**Results:**

Encapsulation efficiencies of Alg-PB beads with 0.5, 1.0, 1.5, and 2.0% polymer solution were 99.9, 91, 92, and 93%, respectively. SEM and particle size analysis revealed differences between formulations in their appearance and size distribution. No drug release was seen in acidic media (pH of 1-2) while complete release was observed at pH of 6.8. Dissolution data was fitted to various mathematical models and beads were found to follow Hixson-Crowell mechanism of release. The pH-dependent release of beads was confirmed* in vivo* by pharmacoscintigraphy in humans.

## 1. Introduction 

Prussian blue (ferric hexacyanoferrate) is a dye used for the internal decorporation of Cesium-137 (^137^Cs) and Thallium-201 (^201^Tl) [1, 2]. It is a crystal lattice that exchanges potassium for cesium at the surface of the crystal [3]. Prussian blue (PB) can be administered in two different physicochemical forms, namely, soluble (colloidal) and insoluble (noncolloidal), both being equally effective* in vivo *[2, 4]. Differences in quality of preparation, particle size, and local pH affect cesium adsorption to the PB crystal lattice and may explain some of the variability in its efficacy seen among patients [5]. On the basis of its very high affinity for cesium and thallium, US-FDA has approved insoluble PB for the removal of ^137^Cs and ^201^Tl. These ions are ordinarily excreted into the intestine and reabsorbed from the gut into the blood and then excreted again into the GI tract. Orally administered PB traps thallium or cesium in the gut, interrupts its reabsorption from the gastrointestinal tract and thereby increasing its excretion through feces. Thus, the biological half-life of thallium and cesium is significantly reduced after decorporation therapy with PB. PB itself is not absorbed across the gut wall in significant amounts [6, 7].

Insoluble PB has been used both in animals and clinically in humans, including during the Goiania incident in Brazil, in which 104 individuals showed evidence of internal contamination with radiocesium [8, 9]. Analysis of the data with respect to PB therapy has revealed that increasing PB dose did not lead to any appreciable decrease in the biological half-life of ^137^Cs [10]. Also, pH has been reported to be a major factor that affects the efficacy of PB* in vivo *[11].

Since PB is a costly drug and may not be easily available as a pharmaceutical grade chemical, it was thought worthwhile to design and evaluate a pH-dependent release oral dosage form of PB. Moreover, since the site of action of PB is intestines, the aim was to release PB directly in the intestine, whereby the drug is delivered at the exact site of action, giving rise to a possibility of reducing the dose in comparison to the present regime wherein a lot of drug gets wasted in the stomach milieu [2, 6].

In the present study, we have prepared pH-dependent oral delivery system of PB by using alginate as a polymer. Alginate has been routinely used in biomedical applications because of its nontoxic and biodegradable nature when given orally [12, 13]. Alginate gel beads are commonly obtained by dropping solutions of sodium alginate into solutions of calcium chloride [1, 14]. These gel beads shrink at acidic pH and get eroded at alkaline pH. Therefore, these can be used effectively to deliver drugs in the intestine, which has a pH of >6.7. Using the same principle, we have prepared beads of calcium alginate-Prussian blue (Alg-PB) in different ratios and characterized them for encapsulation efficiency, surface morphology and in vitro dissolution profile. pH-dependent release of Alg-PB beads was studied at pH of 3, 4, 5, 6, and 7 in vitro. The adsorption efficacy of Alg-PB beads for ^201^Tl was evaluated and compared with native PB.* In vivo* pH-dependent release characteristic of the formulation was studied in humans using gamma scintigraphy. Thus pH-dependent release delivery system of PB was developed and evaluated both in vitro and* in vivo*.

## 2. Material and Methods

The drug substance, Prussian blue (ferric hexacyanoferrate), was procured from John Bakers, Colorado, USA. Sodium alginate was purchased from Loba Chemie, Bombay, India. Sodium hydroxide, sulphuric acid, and other reagents of analytical grade were purchased from local suppliers.

### 2.1. Preparation and Characterization of Alginate Gel Beads

One gram PB was added to 10 ml of sodium alginate aqueous solution of varying concentration, namely, 0.5, 1.0, 1.5, or 2.0% w/v, respectively, so as to select the final drug: polymer ratio depending upon the desired release characteristics. Each solution was mixed and vortexed thoroughly for 5–10 min. The solution was then dropped through a 21 G needle into 10% w/w CaCl_2_ solution, which was being continuously stirred. These droplets of the mixture formed gel beads instantaneously. Beads were allowed to sink, filtered, and washed 2-3 times with distilled water before drying at room temperature for 12 h. Surface morphology of dried hydrogel beads was determined by scanning electron microscopy (Jeol JSM-840).

### 2.2. Drug Loading and Encapsulation Efficiency

One mg of powdered beads was dissolved in 1 ml of 4 N NaOH. 200 *μ*L of concentrated sulphuric acid was added after 10 min of incubation. The volume was made up to 10 ml with distilled water and absorbance was taken at 710 nm as per previously reported method [15]. Concentration of PB was read from the absorbance versus concentration standard plot of PB and hence the PB content was calculated in 1 mg beads. The encapsulation and loading efficiency were calculated as follows:

(1) Drug  loaded=Drug  content calculated×100Wt  of  the  beadsEncapsulation  efficiency=Drug  content calculated×100Amount  of  drug  added.

### 2.3. SEM Studies of Beads

Scanning electron microscopy (SEM) was carried out to study the surface morphology of beads. Samples were mounted on an aluminum stage using adhesive carbon tape and placed in a low humidity chamber for 12 h prior to analysis. Samples were coated with gold-palladium for 60 s under an argon atmosphere using an Emscope® Model 500 Sputter Coater in a high vacuum evaporator equipped with an omnirotary stage tray. SEM was performed using a Jeol JSM-840 scanning microscope (Jeol, Ltd.) operating at an accelerating voltage of 10 kV and a 30 *μ*A and 29 *μ*m emission current. Images were captured with Quartz® software. It was used to evaluate surface texture, shape, and average size of particles. The cross-sectional view of the beads was also studied using SEM.

### 2.4. Release Studies

In vitro drug release studies were performed in triplicate for each of the four test formulations using the USP XXIII dissolution apparatus Type 2 (Scientific Systems 8S) at 75 rpm as per International Conference on Harmonization (ICH) guidelines [16]. The media used were 500 ml deaerated 0.1 M HCl (pH of 1.2), which was maintained at 37°C for first 2 h and then replaced with pH 6.8 phosphate buffer. Approximately, 0.5 g beads were used for each experiment. Samples (5 ml) were taken at different time intervals and assayed spectrophotometrically at 710 nm as described in previous section. At each time of withdrawal, 5 ml of fresh medium was replaced into the dissolution apparatus to maintain sink condition.

In another set of experiments, pH dependence of release was studied in citrate-phosphate buffers of pH 3, 4, 5, 6, and 7. 0.5 g of 1% PB-Alg beads were subjected to dissolution studies as described above and concentration of PB was determined as per the method described by Nagaraja et al. [15].

### 2.5. Analysis of Release Mechanism

The release mechanism of PB from 0.5, 1, 1.5, and 2% Alg-PB beads was examined in accordance with the kinetic models. The regression coefficient, *R*^2^ value nearer to 1.0, indicated the model fitting of the release mechanism. The commonly adopted models for understanding the release of drugs from matrices, namely, zero-order equation, first-order equation [17, 18], Higuchi equation [19], Hixson-Crowell equation [20], Weibull equation [21], and Korsmeyer-Peppas simple exponential equation [22, 23], were used to elucidate the mode of release using SigmaPlot TM-10 software (Cranes Software International, Bangalore, India).

### 2.6. In Vitro Binding Efficacy of Alg-PB Beads

Three sets of 1 mg of the powdered Alg-PB beads' formulations A, B, C, and D each were incubated with 10 MBq ^201^Tl in 5 ml of saline. 5 ml saline (pH of 6-7) having 10 MBq ^201^Tl was used as negative control and 1 mg native PB in saline containing 10 MBq ^201^Tl was used as positive control. 500 *μ*l of the sample was withdrawn from each after 1 h (equilibrium time) and centrifuged [2]. Radioactivity was measured in 100 *μ*l of the supernatant using a well type gamma ray spectrometer (ECIL, India). Percentage adsorption was calculated by dividing the radioactive counts of supernatant of the test by that of negative control.

### 2.7. Pharmacoscintigraphy Evaluation

*In vivo* pH-dependent release of Alg-PB beads was studied in healthy human volunteers by gamma scintigraphy using technetium pertechnetate (^99m^TcO_4_^−^). The study protocol was approved by the Institutional Human Ethics Committee duly constituted for the purpose vide letter no. INM/TS/IEC/005/07.

#### 2.7.1. Preparation of Radiolabeled Beads

Beads were prepared containing 37 MBq ^99m^TcO_4_^−^ and washed thoroughly with distilled water before drying. 1% w/v alginate solution containing 1 g of PB and ^99m^TcO_4_^−^ was dropped in 10% w/v CaCl_2_ solution of 1 N HCl. The beads were formed and separated by filtration. The beads were washed thoroughly with distilled water to remove adhering pertechnetate ions and dried at room temperature for 12 h. In vitro release kinetics of radioactivity were studied as described in Section 2.4.

#### 2.7.2. Release Profile of Labeled Dosage Forms

In vitro release profile of radiolabeled dosage form was determined by measuring radioactive counts released at regular time intervals of 15 min till 3 h. 0.1 N HCl was used as dissolution media (Scientific Systems 8S) for first 2 h followed by phosphate buffer of pH 6.8. The study was carried out at 75 rpm and 37°C. 1 ml samples were withdrawn and measured for radioactive counts in gamma ray spectrometer (ECIL, India). The release pattern of activity was compared to the drug release.

#### 2.7.3. Gamma Scintigraphy

Three healthy human volunteers were given orally 200 mg of ^99m^Tc-labeled Alg-PB beads having 37 MBq ^99m^TcO_4_^−^. In control group, 37 MBq of liquid ^99m^TcO_4_^−^ was administered orally. 64 frames each of 30 sec dynamic images were taken of the abdominal area. Blood pool activity of control and the test subjects was monitored with respect to GIT transit of beads. A region of interest was marked over liver and counts/sec versus time graph was plotted using software eNTEGRA version 2.5.

### 2.8. Statistical Analysis

Data are expressed as mean ± SD. Unpaired *t*-test was applied for the calculation of significance at *p* < 0.05 using GraphPad Instat version 3.00 for Windows XP, GraphPad Software, San Diego, California, USA.

## 3. Results and Discussion

### 3.1. Drug Loading and Encapsulation Efficiency

Table 1 represents the data with respect to drug loading and encapsulation efficiency of PB loaded alginate beads. An encapsulation efficiency of 99.89%, 91.03%, 92.58%, and 93.69% was achieved with 0.5, 1.0, 1.5, and 2.0% polymer solution, respectively, for PB. However, an increase in polymer content inversely affected the percentage of drug loaded with 94.9, 81.93, 78.69, and 74.96% of the drug being loaded (w/w) in case of 0.5%, 1%, 1.5%, and 2% alginate conc., respectively. Using 0.5% alginate conc. resulted in beads being fragile, with a high degree of wear and tear even while preparing the sample. On the other hand, 1% PB-Alg beads not only exhibited good encapsulation efficiency for Prussian blue, but proper beads were also formed. Since pourability of 1.5% and 2% PB-alginate suspensions was poor as compared to 1% PB-alginate suspension; therefore, 1% PB-Alg beads were selected as the final formulation for further studies.

### 3.2. Surface Morphology

**Figure 1:**
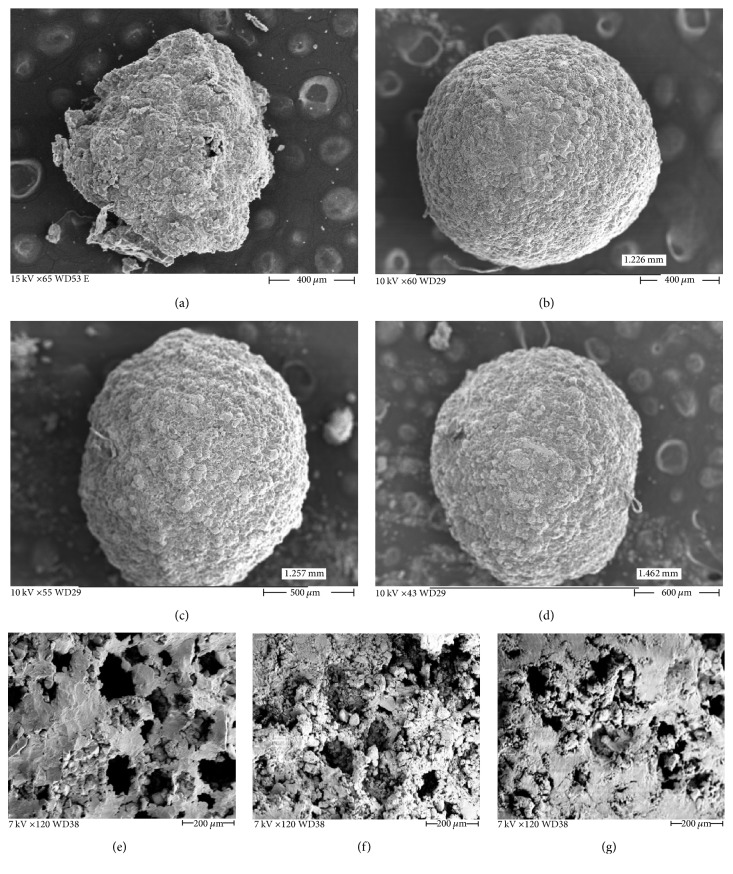
SEM micrographs of (a) 0.5%, (b) 1%, (c) 1.5%, and (d) 2% PB-Alg beads and cross-sectional view of (e) 1%, (f) 1.5%, and (g) 2% PB-Alg beads.

The PB-alginate beads were characterized for their morphological properties by scanning electron microscopy (SEM). In order to investigate the morphology and gain insight into the PB-Alg beads, SEM analysis of void alginate beads and PB encapsulated alginate beads was studied. The diameter of beads varied from 1.2 to 1.5 mm as the alginate concentration was increased from 1 to 2%. The 0.5% polymer beads as observed under SEM were found to be fragile and had a high degree of wear and tear even while preparing the sample (Figure 1(a)). 1% alginate polymer beads were found to be optimal because they had sufficient strength and intactness and maximum PB content of 91% could be incorporated into them. 1.5 and 2% beads were a bit harder and the pourability of polymer solution containing drug through 21 G needle was poor in their cases. However, no significant difference was observed in surface morphology of beads having 1, 1.5, and 2% polymer concentration (Figures 1(a)–1(d)). From the cross-sectional view of the beads under SEM, it was observed that the porosity decreased with increase in polymer concentration (Figures 1(e)–1(g)).

Surface morphologies of drug loaded 1% polymer beads and void beads were also compared. PB loaded beads had rugged surface whereas void beads had comparatively far smoother surface. No pores were visible in the void beads whereas PB loaded beads were porous. The pores present in drug loaded beads may help in fast release of PB at pH 6.8. However, void alginate beads were hard and did not erode easily.

### 3.3. Release Studies

**Figure 2:**
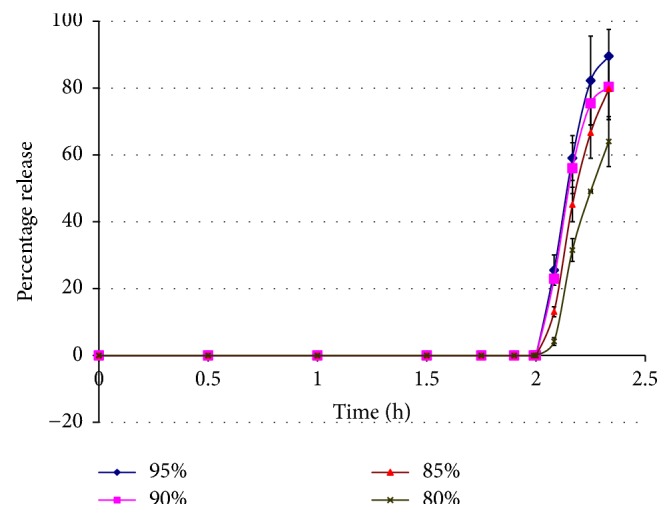
Cumulative percent release of PB from Alg-PB beads. Error bars represent standard deviation about the means based on 3 × 6 replicates. The pH of dissolution media is 1-2 till 2 hrs. Thereafter pH of the dissolution media changes to 6.8, wherein PB gets released immediately.

Despite their different compositions, the beads having varying concentration of sodium alginate [0.5% (A), 1% (B), 1.5% (C), and 2% (D)] and PB showed more or less similar release behavior (Figure 2). The pH-dependent release studies of PB encapsulated in alginate beads were conducted in 0.1 N HCl for the first 2 hours followed by a different dissolution media, that is, phosphate buffer of pH 6.8. In all cases a pH-dependent drug release was seen. For the first 2 hours of dissolution in 0.1 N HCl, no PB was released. With the change in dissolution media to phosphate buffer of pH 6.8, in next 5 min, 31%, 26.57%, 13.08%, and 7.48% of PB was released, respectively, in case of A, B, C, and D whereas complete drug got released within 30 min in all the four formulations. There was little difference in release kinetics of all the formulations, indicating a very mild influence of difference in sodium alginate concentration.

**Figure 3:**
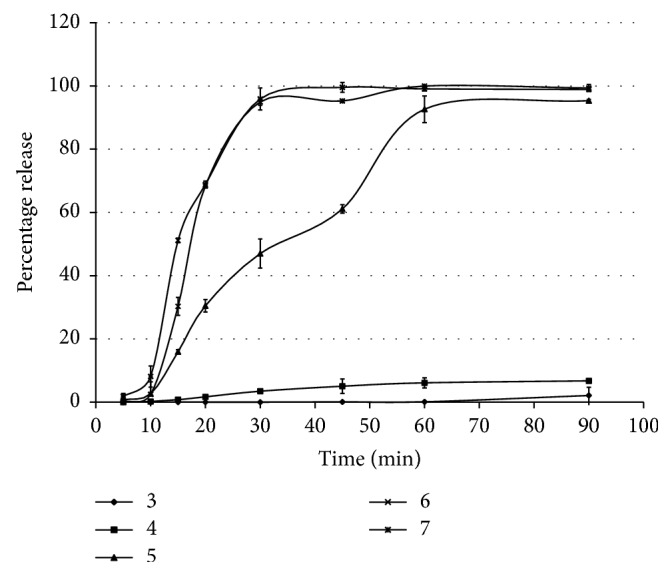
PB release from 1% Alg-PB beads at pH of 3, 4, 5, 6, and 7. Error bars represent standard deviation about the means based on 3 × 6 replicates.

In the experiment where pH-dependent release of 1% PB-Alg beads was studied using citrate-phosphate buffer of pH of 3, 4, 5, 6, and 7, no PB was released till 30 min at pH 3 and only 0.3% release was observed after 90 min. Even at pH 4 only 6.27% of the drug was released at the end of 90 min. The rate of release of the drug at pH 5 was slower than pH of 6 and 7. Approximately 95% of the drug got released at pH of 6 and 7 within 30 min. This further corroborates our observation of release of PB in pH 6.8 phosphate buffer. The results obtained confirm that the beads showed a pH-dependent release pattern (Figure 3).

Although many other pH-dependent eroding polymers in varying conc., namely, hydroxy propyl methylcellulose phthalate (HPMCP), cellulose acetate phthalate, and eudragit, were tried, none of them was able to achieve the desired pH-dependent release of PB. For example, while use of 2.5 and 5% w/w of HPMCP showed complete release of PB in acidic media, not only did 7.5 and 10% w/w HPMCP matrix tablets of PB restrict the release of drug in acidic pH, they also did the same at pH 6.8, thereby defeating the very objective of undertaking this work.

Sodium alginate was chosen for its ability to form a gel/meshwork in the presence of divalent cations such as CaCl_2_, which shrinks at acidic pH and erodes at alkaline pH. Sodium alginate has therefore been used effectively to deliver drugs to the intestine, which has a pH of >6.7 [24, 25]. Moreover, alginate is mucoadhesive and is likely to stick to intestinal mucosa for prolonged periods of time, thereby increasing the time for which the formulation will be available for local action [26, 27]. Further, alginates have also been reported to bind strontium, though no effort was made in the present study to validate this observation [28].

### 3.4. Analysis of Release Mechanism

Table 2 gives the mean correlation coefficient and release constants of various kinetic models. Hixson-Crowell mechanism of release was found the best fit model for explaining the release of PB from alginate beads. The graphic of the cubic root of the unreleased fraction of PB versus time was found to be linear and the correlation coefficients for all the systems (A to D) were approaching 1, suggesting that Alg-PB beads were following Hixson-Crowell mechanism of release, with the drug being released from Alg-PB beads as they were getting eroded with time. The data fitted to the Hixson-Crowell equation showed a correlation coefficient of 0.991 to 0.997 in all the four formulations and *K* value showed a decreasing order with increase in polymer content. According to Hixson-Crowell model, geometrical shape of the dosage form diminishes with time until equilibrium is achieved and the release rate is limited by drug particles' dissolution rate and not by diffusion that might occur through the polymeric matrix [29]. Moreover, PB being an insoluble drug, therefore the drug could not get released by diffusion through the polymeric matrix.

### 3.5. In Vitro Binding Efficacy for ^201^Tl

**Figure 4:**
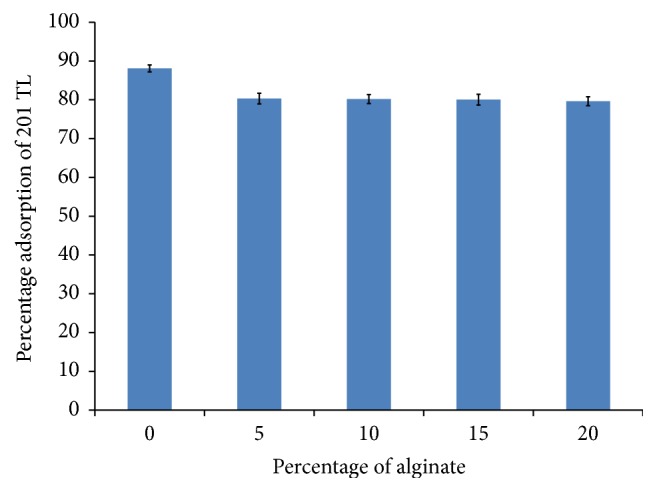
Adsorption efficiency of Alg-PB beads for ^201^Tl. Error bars represent standard deviation about the means based on three replicates.

PB has been used both in animals and in humans as antidote in case of thallium poisoning. US-FDA has accorded its approval to PB to be used for the removal of ^137^Cs and ^201^Tl [6]. Therefore, in order to study the efficacy of PB incorporated in alginate bead matrix, powdered Alg-PB formulations A, B, C, and D were incubated with ^201^Tl for a period of 1 h as per previously reported method from our laboratory [2]. The results of this experiment showed 88.26 ± 1.51% adsorption of ^201^Tl by 1 mg of native PB and 82.66, 82.37, 81.33, and 80.73% adsorption by 1 mg of powdered formulations A, B, C, and D, respectively (Figure 4). The results thus obtained indicate that the binding efficacy of PB does not significantly reduce after its incorporation in the calcium alginate matrix.

### 3.6. Pharmacoscintigraphy Evaluation

**Figure 5:**
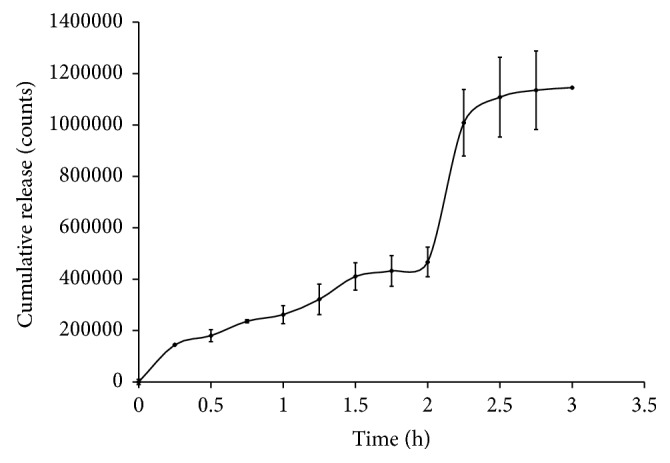
Release profile of radioactivity (technetium-99m pertechnetate, ^99m^TcO_4_^−^) from ^99m^TcO_4_^−^ loaded Alg-PB beads.

**Figure 6:**
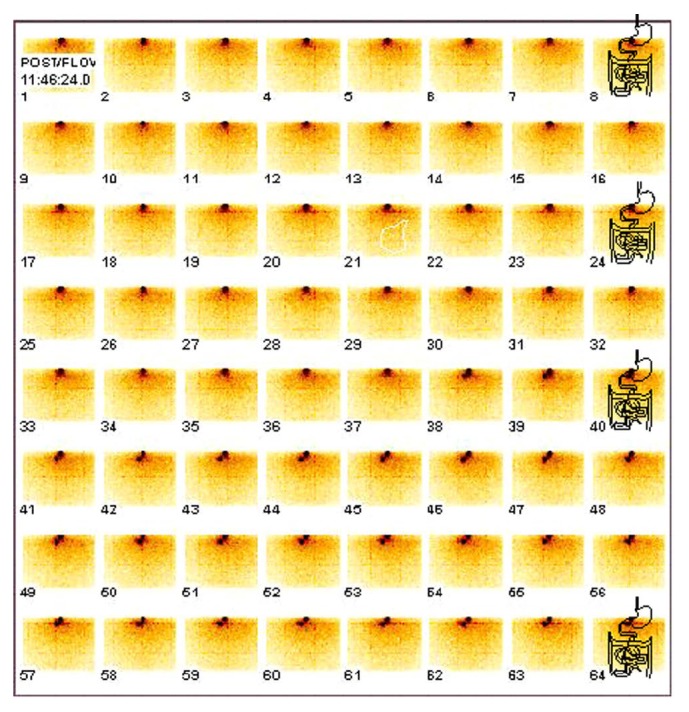
Image showing 32-minute continuous dynamic imaging of subject 1. Gamma camera was focused on lower abdomen and intestines. Each frame lasted for 30 sec and the movements of beads were observed in 38th frame (at 19 min).

**Figure 7:**
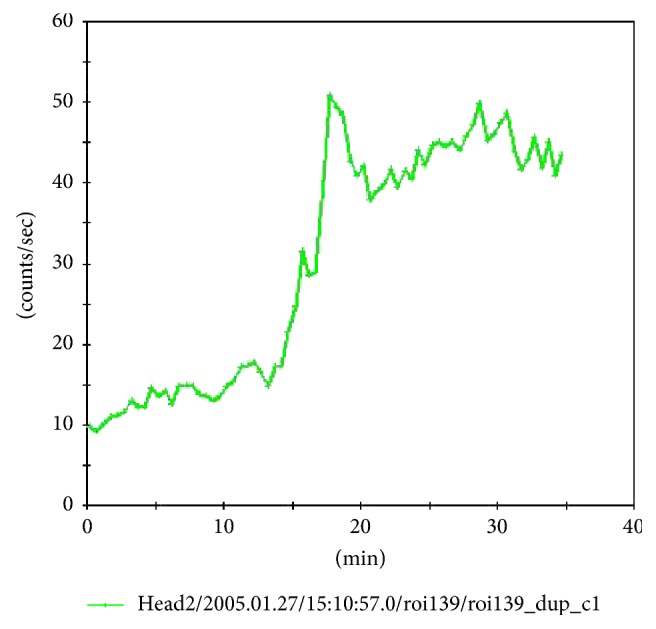
Graph showing the blood pool activity as counts per sec as a function of time.

Release profile of radioactivity (^99m^TcO_4_^−^) from technetium pertechnetate loaded Alg-PB beads showed a biphasic release curve when percentage cumulative counts released were plotted against time (Figure 5). The first phase was a slow release phase in acidic pH of 1-2. Approximately 40% of total radioactivity was released in acidic media till 2 h and the rest was released within next 30 min in phosphate buffer of pH 6.8. However, no release of PB was observed in acidic media, but free pertechnetate being water soluble and a small moiety diffused out of the alginate bead matrix in acidic media of pH of 1-2 with a very slow rate. At higher pH of 6.8, the rate of release was faster and 70% activity got released within 70 min. Therefore, assuming that when radiolabeled beads are in stomach, slow release of radioactivity will occur and as free pertechnetate is released in stomach by the formulation, it will be immediately absorbed into the systemic circulation [30]. Similarly, as the beads will enter intestines, due to the higher pH, beads will erode and free pertechnetate will be released at a faster rate. Thus, the blood pool activity will increase sharply. With this concept, pH-dependent release of Alg-PB was studied qualitatively in humans by monitoring the blood pool activity with respect to position of beads in the GIT. Scintigraphic images were acquired immediately after administration of beads and 80 frames each of 30 sec were taken. Scintigrams of radiolabeled Alg-PB beads in test subject showed the movement of beads in intestine after 20 min (Figure 6). Figure 6 is a 64-frame dynamic gamma scintigraphy image of a human subject who has been given 200 mg of ^99m^Tc-labeled Alg-PB beads having 37 MBq ^99m^TcO_4_. Each frame is of 30 sec each. The dark spots in the frames are the ^99m^Tc-labeled Alg-PB beads. These beads also contain some loosely bound, untrapped ^99m^TcO_4_ in free form, which gets released from these beads immediately and can be seen in the blood pool as tiny spots spread over in each frame. Blood pool activity was monitored by making region of interest (ROI) over liver and heart, and radioactivity of ROI was plotted as counts/sec versus time and shown subsequently in Figure 7, which shows a biphasic release. First phase existed till 20 min, until the time beads were in stomach. Second rapid release phase started after 20 min indicative of the release in intestine. First phase was found to be a slow release phase in which little amount of activity got absorbed in blood. On the other hand, after 20 min, blood pool activity increased very sharply. The slope of the two phases was visually different. For control subjects who were only given 37 MBq of liquid ^99m^TcO_4_^−^ orally and not given PB-Alg beads treatment, dynamic gamma scintigraphy images were not acquired because it is already reported that in such cases ^99m^TcO_4_^−^ gets absorbed immediately and blood pool activity reaches very high levels within 2 min time [30]. These findings corroborated our in vitro release profile.

In vitro and* in vivo* release profiles of radiolabeled Alg-PB beads were found to be superimposed. Both have a first slow release phase, suggesting that some amount of free pertechnetate leaches out of beads even at acidic pH. As the beads come in contact with basic pH of 6.8 phosphate buffer in vitro and basic pH of intestines (duodenum), Alg-PB beads erode and entire amount of free pertechnetate is released rapidly.

Prussian blue (PB) is an emergency/disaster management drug and not much of the related work has been done of it in the past. Post-Fukushima incident, there has been a renewed interest in developing strategies for radioactive cesium decontamination and decorporation using PB. However, much of the work reported for PB in last few years involves remediation of Cs-137 contaminated environments, primarily radiocontaminated water [31–35]. There is limited published literature on developing/improving countermeasures for internal decorporation of cesium from the body. The only formulation of Prussian blue approved by US-FDA for internal decorporation is Radiogardase®. Research groups from across the world are now trying to apply latest pharmaceutics principles to improve this conventional release capsule formulation, so as to improve its efficacy. Some efforts have been made in this direction, wherein preclinical studies in mouse model have been performed [36].

The present study has established the pH-dependent delivery of PB from calcium alginate gel beads both in vitro and* in vivo*. Calcium alginate beads behaved as an erodible matrix, which at higher pH eroded to release PB. Insignificant change in the adsorption efficacy of loaded and native PB was found during in vitro studies with ^201^Tl.* In vivo* pharmacoscintigraphic evaluation in healthy human volunteers proved the pH-dependent release of the drug from PB-Alg beads. Thus, this study shows that the developed formulation can be used to achieve the intestinal release of PB, with a possibility of increasing its efficacy at a lower dose.

## Figures and Tables

**Table 1:** Drug loading and encapsulation efficiency of PB loaded alginate beads.

	Formulation			
	A	B	C	D
Percentage of sodium alginate solution	0.5%	1%	1.5%	2%
(1) Drug loaded (% w/w)	94.90 ± 0.96	81.93 ± 0.39	78.69 ± 1.07	74.96 ± 0.18
(2) Encapsulation efficiency	99.89 ± 1.02	91.03 ± 0.45	92.58 ± 0.31	93.69 ± 0.60

**Table 2:** Comparison of results of the fit to various release models.

Release models		A	B	C	D
Zero order	*K*	0.042 ± 0.007	0.042 ± 0.004	0.047 ± 0.004	0.051 ± 0.005
	*R* ^2^	0.863 ± 0.103	0.842 ± 0.102	0.947 ± 0.019	0.951 ± 0.028

First order	*K*	0.22 ± 0.095	0.163 ± 0.058	0.126 ± 0.047	0.126 ± 0.041
	*R* ^2^	0.971 ± 0.040	0.949 ± 0.041	0.995 ± 0.001	0.974 ± 0.023

Higuchi	*K*	0.318 ± 0.03	0.294 ± 0.028	0.309 ± 0.030	0.345 ± 0.033
	*R* ^2^	0.9502 ± 0.02595	0.902 ± 0.083	0.974 ± 0.007	0.993 ± 0.001

Hixson-Crowell	*K*	0.299 ± 0.121	0.249 ± 0.062	0.218 ± 0.056	0.220 ± 0.051
	*R* ^2^	0.998 ± 0.007	0.997 ± 0.043	0.993 ± 0.006	0.991 ± 0.009

Weibull Equation	*b*	1.687 ± 0.271	1.755 ± 0.216	1.970 ± 0.159	2.379 ± 0.300
	Td	8.955 ± 0.027	9.529 ± 0.216	13.417 ± 0.159	14.356 ± 0.300
	*R* ^2^	0.985 ± 0.006	0.985 ± 0.024	0.979 ± 0.024	0.979 ± 0.015

Korsmeyer-Peppas	*K*	0.16 ± 0.057	0.047 ± 0.031	0.009 ± 0.047	0.003 ± 0.048
	*R* ^2^	0.994 ± 0.007	0.959 ± 0.042	0.968 ± 0.021	0.965 ± 0.021
	*n*	1.082 ± 0.024	1.111 ± 0.044	1.667 ± 0.358	2.133 ± 0.395

*K*, release rate constants; *R*^2^, correlation coefficient; *n*, exponent release; Td, lag time measured as a result of the dissolution process; *b*, shape parameter of formulation.
